# Determination of μmol l^-1^ level of iron (III) in natural waters and total iron in drugs by flow injection spectrophotometry

**DOI:** 10.1155/S1463924699000024

**Published:** 1999

**Authors:** B. Sahasrabuddhey, S. Mishra, A. Jain, K. K. Verma

**Affiliations:** Department of Chemistry Rani Durgavati University Pradesh Jabalpur Madhya 482001 India

## Abstract

The equilibrium problems, characterized by recurring end-points, involved in the reaction of iron (III) with iodide make the batch iodometric determination of iron (III) unsuitable. Since the flow injection determination does not require attainment of steady state either for mixing of reagents or for the chemical reaction, the iodometric determination has been accurately and precisely performed using this technique in the present work. This method does not require any special reagent, including chelating agents or those which are loxic, and has a limit of detection of 0.2 μmol l^-1^ (11 μg l^-1^) of iron (III). The interference of fluoride has been avoided by adding zirconyl nitrate to the test sample solution, and of copper (II) by complex formation with 2-mercaptobenzoxazole. The method has been applied to determine iron (III) in natural waters, and total iron in drugs.


Journal of Automated Methods & Management in Chemistry, Vol. 21, No. 1 (January–February 1999) pp. 11–15

### Determination of $\mu mol\,l^{-1}$ level of iron (III) in natural waters and total iron in drugs by flow injection spectrophotometry

**B. Sahasrabuddhey, S. Mishra, A. Jain and K. K. Verma**

*Department of Chemistry, Rani Durgavati University, Jabalpur 482001, Madhya Pradesh, India*

*The equilibrium problems, characterized by recurring end-points, involved in the reaction of iron (III) with iodide make the batch iodometric determination of iron (III) unsuitable. Since the flow injection determination does not require attainment of steady state either for mixing of reagents or for the chemical reaction, the iodometric determination has been accurately and precisely performed using this technique in the present work. This method does not require any special reagent, including chelating agents or those which are toxic, and has a limit of detection of 0.2 $\mu mol\ l^{-1}$ (11 $\mu g\ l^{-1}$) of iron (III). The interference of fluoride has been avoided by adding zirconyl nitrate to the test sample solution, and of copper (II) by complex formation with 2-mercaptobenzoxazole. The method has been applied to determine iron (III) in natural waters, and total iron in drugs.*

### Introduction

Iron is an essential trace element involved in normal growth and development. The importance of iron in nutrition has been recognized and, therefore, this element is often added in certain foodstuffs. Reliable speciation of iron (II) and iron (III) is fundamental for the proper characterization of many of the processes in terrestrial and aquatic environments [1], and is also of practical importance in the investigation of corrosion of iron and the treatment of wastewaters from the mining and steel industries [2]. The determination of the oxidation state of iron in a variety of natural water samples has generally been performed by complexation with specific chelating agents followed by spectrophotometry [3–8] or voltammetry [9–11]. To attain adequate sensitivity of the method in dealing with the analysis of iron at $\mu mol\,l^{-1}$ or lower levels, pre-concentration procedures have been reported using the natural polymer Chitin [12], Chelex 100 [13], melamine-formaldehyde resin [14], or by solid phase extraction on $C_{18}$ cartridge [15,16].

When a chelating agent is developed for determination, one has to be aware of the strength of the iron–chelator complex. This is important in order to assess the usefulness of the chelator with respect to naturally occurring chelators in the ambient water samples. If naturally occurring ligands have large stability constants, e.g. EDTA–iron (III) ($\beta = 10^{25}$), then the concentrations obtained with chelating methods are only erroneous [8]. Another aspect deals with the potential changes in the oxidation state of iron in the sample due to the changes in the redox potential between iron (II)/iron (III) oxidation states. This change in redox potential is due to the favourable stabilization of one oxidation state over the other when a chelating agent is added to the natural water samples. This is an important aspect in measuring a specific redox state of iron at low ambient levels. Thus, techniques which eliminate the need for using chelating agents are worth re-evaluation.

In the present work, a flow injection spectrophotometric method is described which does not make use of chelating agent or toxic reagents, but is sensitive to $\mu mol\,l^{-1}$ levels of iron (III).

### Experimental

### *Instrumentation*

Shimadzu LC-5A reciprocating pump (Tokyo) and Ismatec Mini-S 820 peristaltic pump (Zurich) were used for propelling the carrier stream of water and reagents, respectively. Rheodyne 5020 low pressure PTFE 4-way valve (Anachem, Luton) was used for injection of sample solution. Shimadzu SPD-2A variable wavelength detector (8 $\mu$l flow-through cell) and Shimadzu C-R2AX integrator fitted with a printer was used for measurement of peak height. PTFE tubings (Anachem), 0.5 mm i.d., were used for construction of flow lines. All flanged connections were made with plastic nuts and TEFZEL coupler. Home-made T-joints of 0.8 mm i.d. were employed.

The flow-injection manifold used for the analysis of iron (III) is given in figure 1. It consisted of three channels, the first two, mounted on a peristaltic pump, were used for propelling potassium iodide and hydrochloric acid streams. Both iodide and acid were mixed in a delay coil and downstream merged with the third channel used for the water carrier stream. The four-way loop injection valve was mounted on the water carrier stream. Another delay coil was used for the reaction to occur before detection at 360 nm.

### *Reagents and standards*

Hydrochloric acid, 0.25 $mol\,l^{-1}$ was used as the carrier in the optimized method.

A stock 0.01 $mol\,l^{-1}$ iron (III) solution was prepared as follows. Iron (II) ammonium sulphate, 3.92 g, was dissolved in about 100 ml of water, mixed dropwise while stirring with 5 ml of concentrated nitric acid, boiled for 5 min, and then cooled to 50–60°C. About 3 g of potassium peroxodisulphate was added portionwise with stirring. The solution was diluted to about 200 ml and again

1463-9246/99 $12·00 © 1999 Taylor & Francis Ltd

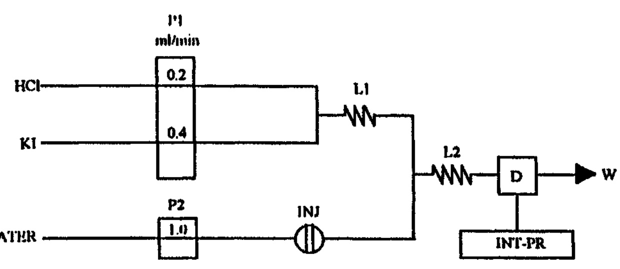

*Figure 1. Schematic diagram of the flow injection manifold used for the determination of iron(III). Potassium iodide, 8%, and 0.25M hydrochloricid acid were used as reagents, and deinized distilled water as carrier. P1 = peristaltic pump; P2 = HPLC reciprocating pump; INJ = injection valve (100-μl loop); L1 = 50 cm mixing coil; L2 = 20 cm reaction coil, D = detector set at 360 nm; INT-PR = integrator and printer; W = waste. All flow lines were made from 0.5 mm i.d. PTFE tubing.*

boiled for about 10 min. The cooled solution was treated with ammonia (1:1, concentrated ammonia–water) till slight precipitation of iron (III) hydroxide. The precipitate was redissolved by adding 0.25 mol $l^{-1}$ hydrochloric acid, and the solution made up to 1 l in a standard flask with the same solvent.

For standardization of iron (III) solution [17,18], a 5 ml aliquot of iron (III) solution was treated either with 5 ml of 0.01 mol $l^{-1}$ ascorbic acid or 10 ml of 0.01 mol $l^{-1}$ mercaptoacetic acid and swirled for 1 min. About 0.1 g of potassium iodide and 1 ml of 1% starch were added, and the residual amount of ascorbic acid or mercaptoacetic acid from the reaction was evaluated by back titration with 0.01 mol $l^{-1}$ chloramine T, a blue colour was obtained at the end-point. Iron (III) reacts with ascorbic acid or mercaptoacetic acid in a molar ratio of 2:1 and 1:1, respectively. The strength of iron (III) solution determined by two methods agreed within 1%.

The stock solution was sequentially diluted with 0.25 mol $l^{-1}$ hydrochloric acid to give test solutions of iron (III).

All other substances used were of high purity, and their solutions were made by dissolving the right amounts in water.

### *Procedures*

*Wet ashing of iron tablets.* A known number of tablets or contents of capsules were weighed and finely ground. A weight equivalent to a tablet or contents of a capsule was transferred to a 100 ml Kjeldahl flask, mixed with about 20 ml of water and boiled over a small flame till the volume was reduced to about 5 ml. The solution was cooled to room temperature and carefully mixed with 15 ml of concentrated nitric acid and then with 10 ml of concentrated sulphuric acid. The flask was kept at room temperature for about 15 min then heated slowly to boil the contents under a fume-cupboard where copious fumes of nitrogen oxides evolved. The above process was repeated twice by adding both acids, and heating till no more brown fumes evolved. About 50 ml of water along with 5 g of potassium peroxodisulphate was added, and boiling continued till the contents of the flask evaporated to almost dry. The dried matter was boiled with 25 ml of water, and transferred quantitatively to a 250 ml beaker, filtering any insoluble matter. The clear filtrate was treated with 1:1 ammonia–water till slight precipitation of iron (III) hydroxide, the precipitate was redissolved by adding 0.25 mol $l^{-1}$ hydrochloric acid, and finally diluted to 250 ml in a standard flask with the same solvent.

*Removal of copper (II).* 2-Mercaptobenzoxazole was impregnated on silica gel using the procedure as reported for impregnation of 2-mercaptobenzothiazole [19]. The column was filled with impregnated silica gel to give a bed height of about 1 cm which was secured in position by placing one Whatman filter No. 42 disc above and another below the bed. A known volume of drug solution prepared as above was passed through the column under mild suction (1–2 ml $min^{-1}$). The column was washed with about 2 ml of water. The combined eluent and washings were boiled with 2 ml of concentrated nitric acid, cooled and diluted to a known volume.

### Results and discussion

Though iodimetric titration of iron (III), which involves the reaction of iron (III) with acidified iodide and titration of the liberated iodine with standard thiosulphate, has been recommended in British Pharmacopoeia, 1968, the most frequent [20] difficulties are encountered due to recurring end-points, as the reaction is very slow near the stoichiometric end-point. Extraction of the liberated iodine into chloroform or carbon tetrachloride has been recommended to push the reaction to completion [20]. However, such titrations involving two immiscible phases are always cumbersome and the end-point is often carried over.

Flow injection analysis does not require the attainment of a steady state for mixing of reactants or for the chemical reaction, and the signals, though transient, are highly reproducible. This unique feature of the flow injection technique was tested for the analysis of iron (III) by its reaction with iodide, and the conditions have been optimized for achieving maximum sensitivity.

### *Study of flow injection variables*

The flow injection manifold, as given in figure 1, was used when the acid and potassium iodide reagents streams were each maintained at 0.2 ml $min^{-1}$, carrier flow rate was 1 ml $min^{-1}$, loop size was 75 μl, mixing coil and reaction coil were, respectively, 50 and 25 cm. The effect of all the variables was evaluated. There was an optimum peak height observed when 0.25 mol $l^{-1}$ hydrochloric acid was used. The peak height increased when up to 8% potassium iodide was used, then remained practically constant at higher concentration. In the optimized procedure, an 8% potassium iodide was used. Optimum flow rates were: carrier stream 1 ml $min^{-1}$, acid stream 0.2 ml $min^{-1}$ and potassium iodide stream 0.4 ml $min^{-1}$.

A 50 cm coil was necessary for complete mixing of potassium iodide and hydrochloric acid mixed reagents streams. A 20 cm reaction coil was found to give optimum peak height. The signal was optimum when a 100 μl sample was introduced into the flow system.

*Validation of the analytical procedures*

The flow injection analysis of iron (III) was validated by injecting known amounts of standards in the flow system. Calibration graphs were constructed on two concentration ranges of 0.01–0.1 mmol $l^{-1}$ and 1–10 μmol $l^{-1}$ iron (III). The analytical characteristics of calibration graphs are given in table 1. The limit of detection has been found to be 0.2 μmol $l^{-1}$ (11 μg $l^{-1}$) iron (III) [S/N = 3; RSD = 3.5%] which compares favourably with that of diverse procedures for iron determination (table 2), and is an important feature of this method since no special reagent is necessary.

*Masking of fluoride in natural waters*

Fluoride forms a stable complex with iron (III), hexafluoroferrate (III), which does not liberate iodine on reaction with iodide. Thus, the interference of fluoride was avoided by adding zirconyl nitrate to the test sample solution when a still more stable complex zirconyl fluoride is formed leaving iron (III) free.

*Table 1. Analytical characteristics of the flow injection spectrophotometric determination of iron (III); the absorbance was recorded at 360 nm with AFS 0.32. Six calibration points of five replicates each were sampled.*

| *Range* | *Slope** | *Intercept** | *r* |
|---|---|---|---|
| 0.01–0.1 mmol $l^{-1}$ | 12 938.3 IU.l $mmol^{-1}$ | −37.31 IU | 0.9996 |
| 1–10 μmol $l^{-1}$ | 11.92 IU.l $μmol^{-1}$ | −5.29 IU | 0.9992 |

*IU = integrator arbitrary units.

*Table 2. Comparison of diverse methods for iron determination (without preconcentration).*

| *Technique* | *Reagent* | *Limit of detection, μmol $l^{-1}$* | *Ref.* |
|---|---|---|---|
| Spectrophotometry | Iodide | 0.2 | This work |
| | 1,10-Phenanthroline | 0.36 | 1 |
| | 1,10-Phenanthroline | 0.2 | 3,5 |
| | Ferrozine | 0.1 | 15 |
| | Ferrozine | 0.2 | 8 |
| | 1-Amino-4-hydroxyanthraquinone | 20 | 22 |
| Spectrofluorimetry | 5-(4-methylphenylazo)-8-Aminoquinoline | 0.18 | 23 |
| Potentiometry | Solochrome Violet RS | 0.25 | 24 |
| | 1,10-Phenanthroline | 0.54 | 25 |
| Flame AAS | | 0.2 | 26 |

*Table 3. Determination of iron (III) and total iron in the presence of copper (II) involving sorption of copper (II) with 2-mercaptobenzoxazole.*

| | | *% Found after extraction of copper (II)* | | | |
|---|---|---|---|---|---|
| *Iron (III) μmol $l^{-1}$ taken* | *Copper (II) μmol $l^{-1}$* | *Iron (III)* | *%RSD (n = 5)* | *Total iron* | *%RSD (n = 5)* |
| 5 | 2 | 96.1 | 1.8 | 99.9 | |
| | 5 | 97.2 | | | |
| | 10 | 98.4 | 2.9 | 100.1 | 2.1 |
| 10 | 2 | | | 98.7 | |
| | 10 | 97.1 | | | |
| | 20 | 98.5 | 2.2 | 99.6 | 2.9 |
| 20 | 2 | 96.2 | | | |
| | 20 | 98.2 | | 99.2 | |
| | 40 | 98.8 | 2.5 | 100.5 | 2.2 |
| 40 | 2 | 95.8 | 2.3 | | |
| | 40 | 97.0 | 2.8 | 98.9 | |
| | 80 | 98.2 | | 101.2 | 2.8 |

*Removal of copper (II) from drug formulations*

Sorption of copper (II) by complexation with thiol reagents loaded on to silica gel has been documented [19]. Since all general thiols also reduce iron (III), attempts were made to complex copper (II) without significant reduction of iron (III). Reagents which were tried include 2-mercaptobenzothiazole, -benzimidazole and -benzoxazole, and triphenylmethanethiol. Triphenylmethanethiol showed minimal reduction of iron (less than 1%) but was too sluggish in the sorption of copper (II). 2-Mercaptobenzoxazole was found to be the best reagent among those tested. Results are given in table 3 for the determination of iron (III) and total iron in the presence of copper. There was a 4–5% reduction of iron (III) on the sorbent, however, this was less appreciable when the copper (II)/iron (III) ratio was more than 1, ostensibly due to greater reactivity of the sorbent towards copper (II). Any iron (II) formed during sorption was reoxidized before analysis of total iron in drugs.

*Application of the method to natural waters*

The present flow injection method was applied to determine iron (III) present in certain natural waters. The samples were filtered through a 0.45 μm membrane filter, the filtrate was acidified with 0.5 ml of concentrated hydrochloric acid per 500 ml of sample, treated with 1% (w/v) zirconyl nitrate to mask fluoride, if also present, and analysed. The method was further validated by standard addition method. The results obtained for the addition of known amounts of iron (III) to natural samples gave an average recovery of 102% with a standard deviation of 5.4% (table 4). The concentration of copper (II) in natural waters is usually low enough so as not to interfere in the determination or iron (III) [21].

*Application of the method to the determination of total iron in drugs*

The present method has been applied to determine total iron in certain pharmaceutical preparations. The results obtained have been compared with those found by a previously checked procedure [18] (table 5). The present method has been found to be rapid and precise.

### Interferences

Chelating methods are usually severely interfered by fluoride, EDTA and phosphate. Many of these agents

*Table 4. Flow injection spectrophotometric determination of iron (III) in natural waters.*

| *Water sample* | *Iron (III) added* $\mu mol\, l^{-1}$ | *Iron (III) found* $\mu mol\, l^{-1}$ | *Recovery (%)** | *%RSD (n = 6)* |
|---|---|---|---|---|
| Well water | 0 | 15.0 | | 4.5 |
| | 10.0 | 26.4 | 114 | 5.0 |
| Tap water No. 1 | 0 | 2.32 | | 6.1 |
| | 5.0 | 7.20 | 98 | 5.9 |
| Tap water No. 2 | 0 | 3.89 | | 7.1 |
| | 5.0 | 9.00 | 102 | 5.6 |
| Ground water No. 1 | 0 | 4.05 | | 5.9 |
| | 10.0 | 13.87 | 98 | 5.3 |
| Ground water No. 2 | 0 | 3.75 | | 7.0 |
| | 5 | 8.55 | 96 | 6.5 |
| Ground water No. 3 | 0 | 70.4 | | 4.3 |
| | 50.0 | 125.0 | 109 | 4.6 |

* The results are the average of six determinations.

*Table 5. Results of the flow injection spectrophotometric determination of total iron in drugs.*

| | *Total iron, mg, per tablet or capsule* | | | |
|---|---|---|---|---|
| *Drug* | *Label claimed* | *Found by present method** | *% RSD* | *Found by comparison method [18]†* |
| Fesovit capsule‡ | 54.0 | 48.4 | 5.6 | 50.0 |
| Autrin capsule§ | 113.9 | 109.3 | 6.2 | 105.0 |
| Haematinic tablet¶ | 25.0 | 21.2 | 5.8 | 20.4 |

* The results are the average of five determinations.

† The results are the average of three determinations.

‡ Also contains ascorbic acid (50 mg), thiamine mononitrate (2 mg), nicotinamide (15 mg), pyridoxine hydrochloride (1 mg), pantothenic acid (calcium salt) (2.5 mg) and amaranth.

§Also contains ascorbic acid (150 mg), cyanocobalamine (15 μg) and folic acid (1.5 mg).

¶ Also contains ascorbic acid (35 mg), calcium citrate (500 mg), vitamin B2 (2 mg), vitamin B6 (1 mg), folic acid (0.3 mg) and vitamin E (1 μg). Each tablet was mixed with 5 mg of copper (II).

either do not interfere in the iodide method or can be masked with suitable reagents.

Chlorine and chlorine dioxide are other oxidizing agents that may be present in drinking water along with iron (III). Their interference can be removed by purging with nitrogen (for chlorine dioxide) or treatment with sodium oxalate (for chlorine) before analysis for iron (III). Nitrite can be decomposed with ammonium chloride in feebly acidic medium.

### Conclusions

The sensitivity of the present method involving oxidation of iodide is comparable to those which use chelating agents, e.g. 1,10-phenanthroline or ferrozine, or based on AAS. Flow injection circumvents the equilibrium problems of the reaction which are commonly observed in batch methods.

The present method does not make use of any special reagent. It is simple and rapid, and suitable for sensitive determination of iron (III) in environmental waters, and total iron in drugs. The method has potential for its application to other samples, e.g. foodstuffs.

### References

1. Pehkonen, S., 1995, *Analyst*, **120**, 2655.

2. Clark, L. J.,1962, *Anal. Chem.*, **34**, 348.

3. Chalk, S. J. and Tyson, J. F., 1994, *Anal. Chem.*, **66**, 660.

4. Hase, U. and Yoshimura, K., 1992, *Analyst*, **117**, 1501.

5. Liu, R. M., Liu, D.-J. and Sun, A.-L., 1992, *Analyst*, **117**, 1767.

6. Kang, S. W., Sakai, T., Ohno, N. and Ida, K., 1992, *Anal. Chim. Acta*, **261**, 197.

7. Yi, Z., Zhuang, G. S., Brown, P. R. and Duce, R. A., 1992, *Anal. Chem.*, **64**, 2826.

8. Pascual-reguera, M. I., Ortega-Carmona, I. and Molina-Diaz, A., 1997, *Talanta*, **44**, 1793.

9. Florence, T. M., 1992, *Analyst*, **117**, 551.

10. Wang, L. Z., Ma, C. S., Zhang, X. L. and Wang, J. G., 1994, *Anal. Lett.*, **27**, 1165.

11. Diaz, M. E. V., Sanchez, J. C. J., Mochon, M. C. and Perez, A. G., 1994, *Analyst*, **119**, 1571.

12. Hoshi, S., Yamada, M., Inoue, S. and Matsubara, M., 1989, *Talanta*, **36**, 606.

13. Rubi, E., Jimenez, M. S., Bauza de Mirabo, F., Forteza, R. and Cerda, V., 1997, *Talanta*, **4**, 553.

14. Filik, H., Ozturk, B. D., Dogutan, M., Gumus, G. and Apak, R., 1997, *Talanta*, **44**, 877.

15. King, D. W., Lin, J. and Kester, D. R., 1991, *Anal. Chim. Acta*, **247**, 125.

16. Nickson, R. A., Hill, S. J. and Worsfold, P. J., 1995, *Anal. Proc.*, **32**, 387.

17. Verma, K. K., 1979, *Talanta*, **26**, 277.

18. Verma, K. K., Gupta, A. K. and Bose, S., 1981, *Farmaco Ed. Pr.*, **36**, 23.

19. Terada, K., Matsumoto, K. and Kimura, H., 1983, *Anal. Chim. Acta*, 1983, **153**, 237.

20. Garratt, D. C., 1964, *The Quantitative Analysis of Drugs* (London: Chapman & Hall), p. 350.

21. Yokota, F. and Abe, S., 1997, *Anal. Commun.*, **34**, 111.

22. Abu-Bakr, M. S., Sedaira, H. and Hashem, E. Y., 1994, *Talanta*, **41**, 1669.

23. Yan, G. F., Shi, G. R. and Liu, Y. M., *Anal. Chim. Acta*, 1992, **264**, 121.

24. Janger, R., Renman, L. and Stefansdottir, S. H., 1993, *Anal. Chim. Acta*, **281**, 305.

25. Hassan, S. S. M. and Marzouk, S. A. M., 1994, *Talanta*, **41**, 891.

26. Cresser, M. S., 1994, *Flame Spectrometry in Environmental Chemical Analysis: A Practical Guide* (Cambridge: The Royal Society of Chemistry), p. 85.

